# Effectiveness and safety of Chinese herbal footbaths as an adjuvant therapy for dysmenorrhea: a systematic review and meta-analysis

**DOI:** 10.3389/fphar.2024.1397359

**Published:** 2024-08-05

**Authors:** Xiaoping Tian, Jingwen Wei, Yijia Zhuang, Xiaoding Lin, Liu Liu, Jun Xia, Wenying Huai, Ying Xiong, Yunhui Chen

**Affiliations:** ^1^ CDUTCM-KEELE Health and Medical Sciences Institute, School of Basic Medical Sciences, School of Acupuncture, Moxibustion, and Tuina, Chengdu University of Traditional Chinese Medicine, Chengdu, China; ^2^ West China Hospital, West China School of Medicine, Sichuan University, Chengdu, China

**Keywords:** Chinese herbal footbaths, dysmenorrhea, randomized controlled trials, therapeutic efficacy and safety, meta-analysis

## Abstract

**Objectives:**

To evaluate the effectiveness and safety of Chinese herbal footbaths (CHF) as an adjunctive therapy in managing dysmenorrhea.

**Methods:**

Ten electronic databases were searched to identify eligible randomized clinical trials (RCTs) from inception until June 2023. Outcome measurements encompassed the total effective rate, visual analog scale (VAS) score of pain intensity, Cox menstrual symptom scale (CMSS) score, symptom score, Traditional Chinese Medicine (TCM) syndrome scale, and any reported adverse events. The methodological quality of the included studies was assessed with the Cochrane collaboration tool. Review Manager 5.3 software was employed for quantitative synthesis, and funnel plots were utilized to evaluate potential reporting bias.

**Results:**

Eighteen RCTs with 1,484 dysmenorrhea patients were included. The aggregated results suggested that the adjunctive CHF could significantly ameliorate dysmenorrhea, as evident from the improved total effective rate [risk ratio (RR) 1.18, 95% confidence interval (CI): 1.12 to 1.23, *P* < 0.00001], VAS (MD 0.88, 95% CI: 0.68 to 1.09, *P* < 0.00001), CMSS (MD 3.61, 95% CI: 2.73 to 4.49, *P* < 0.00001), symptom score (SMD 1.09, 95% CI: 0.64 to 1.53, *P* < 0.00001), and TCM syndrome scale (MD 3.76, 95% CI: 2.53 to 4.99, *P* < 0.0001). In addition, CHF presented fewer adverse events with a better long-term effect (RR 1.34, 95% CI: 1.11 to 1.63, *P* < 0.01) and diminished recurrence rate (RR 0.19, 95% CI: 0.09 to 0.39, *P* < 0.0001).

**Conclusion:**

Current evidence implies that CHF may be an effective and safe adjunctive therapy for patients with dysmenorrhea. However, the methodological quality of the studies included was undesirable, necessitating further verification with more well-designed and high-quality multicenter RCTs.

**Systematic Review Registration:**

https://www.crd.york.ac.uk/PROSPERO/display_record.php?RecordID=188256, identifier registration number.

## Introduction

Dysmenorrhea, marked by cramping and pain in the lower abdomen during or before menstruation, remains a prevalent but disregarded, underdiagnosed, and inadequately treated gynecological issue (Itani et al., 2022; MacGregor et al., 2023). It affects up to 93% of adolescents and an estimated 16%–91% of women of childbearing age (Ju et al., 2014; De Sanctis et al., 2015; Campbell, 2019). This ailment, primary or secondary, may severely impact patients’ daily activities, leading to reduced academic achievements among teenagers and reduced productivity and work performance for adults (Tu et al., 2024). In the United States, dysmenorrhea is responsible for approximately 600 million hours of work lost with two billion dollars of financial cost annually (Iacovides et al., 2015). The primary pharmacological remedies include non-steroidal-anti-inflammatory drugs and hormonal contraceptives, yet about 15% of patients find no relief with these interventions. Moreover, prolonged use may cause adverse events affecting the gastrointestinal, neurological, and cardiovascular systems (Oladosu et al., 2018; Lopes Dias et al., 2019). This situation highlights the necesseity for an increased medical attention and alternative treatment strategies (Tu and Hellman, 2021).

Given these challenges, there has been a growing interest in complementary and alternative therapy over recent years, and a substantial number of patients with dysmenorrhea turning to traditional Chinese medicine (TCM) for solution (Sosorburam et al., 2019; Zhang et al., 2024). Chinese herbal footbaths (CHF), an ancient TCM modality dating back over three millennia, has been utilized in China to address a broad spectrum of health issues, including menstrual symptoms. In the CHF treament, individuals soak their feet and lower legs in a warm herbal concoction for 20–30 min, benefiting from more than just relaxation. This external therapeutic approach cooperates the soothing heat and reflective effects with the healing properties of specific Chinese herbs, prescribed in accordance with individual-oriented TCM pattern differentiations (Chen et al., 2019; Xiao et al., 2021). Despite its longstanding usage, the scientific community recently has conducted an increasing body of randomized controlled trials (RCTs) investigating the effectiveness and safety of CHF for dysmenorrhea management, yet a thorough systematic review and meta-analysis consolidating these findings on the subject remains unreported. Hence, this study aimed to methodically assess the available evidence on the effectiveness and safety of CFH in alleviating dysmenorrhea, yielding potentially valuable information for patients, healthcare providers, and researchers concerned.

## Methods

This meta-analysis were implemented following the guidelines of Cochrane Handbook for Systematic Reviews of Interventions and the Preferred Reporting Items for Systematic Reviews and Meta-Analyses (PRISMA) and using the RevMan software (Version 5.3; the Cochrane Collaboration, NCC, CPH, Denmark). Additionally, the protocol was registered and published on PROSPERO (PROSPERO CRD 42020188256) (Xiao et al., 2021).

### Data source and search strategy

Two independent reviewers (JWW and YX) systematically searched ten electronic databases, including the Web of Science, CIHAHL, PubMed, EMBASE, Cochrane Library, China Biomedical Literature Database (CBM), China National Knowledge Infrastructure (CNKI), Chinese Scientific Journals Database (VIP), Wanfang Database, and the Chinese Biomedical Literature Service System (SinoMed), up to June 2023 without any language restriction to identify eligible studies. Search terms were used in a combination as follows: dysmenorrhea, menstrual pain, painful menstruation, period pain, painful period, menstrual cramps, menstrual disorder, pelvic pain, menstrual cramps, painful menstrual periods, Chinese herbal footbaths, bath, hydrotherapy, herbal bathing, lavipeditum, randomized controlled trial, randomized, randomly, trials, and RCT. Manual searches of reference from retrieved articles were also performed. Discrepancies between reviewers were resolved through consultation with a third reviewer. The search strategy used for PubMed is detailed in Table 1 and underwent necessary adjustments to accommodate the requirements of other databases.

**TABLE 1:** Search strategy for the PubMed.

No.	Search terms
#1	dysmenorrhea
#2	menstrual pain
#3	painful menstruation
#4	period pain
#5	painful period
#6	cramps
#7	menstrual disorder
#8	pelvic pain
#9	menstrual cramps
#10	painful menstrual periods
#11	#1 OR #2 OR #3 OR #4 OR #5 OR #6 OR #7 OR #8 OR #9 OR #10
#12	Chinese herbal footbaths
#13	bath*
#14	hydrotherapy
#15	herbal bathing
#16	lavipeditum
#17	#12 OR #13 OR #14 OR #15 OR #16
#18	#11 AND #17
#19	randomized controlled trial
#20	randomized
#21	randomly*
#22	trials
#23	RCT
#24	#19 OR #20 OR #21 OR #22 OR #23
#25	#11 AND #18 AND #24

^*^Represent one or more characters of all characters.

### Eliginility criteria

The inclusion criteria, based on the PICOS (patients, intervention, comparator, outcomes, and study design) framework, were pre-specified as: 1) Participants: patients diagnosed with dysmenorrhea of any age, case source, and disease duration and severity; 2) Intervention: CHF, alone or with other treatments; 3) Comparators: basic or conventional medications, other TCM modalities, placebos, or non-intervention; 4) Outcome measurements: primary outcomes of pain relief measured by total effective rate, and secondary outcomes included pain intensity evaluated by validated scales, such as the visual analog scale (VAS) pain intensity score and the Cox menstrual symptom scale (CMSS) score symptom score, TCM syndrome scale, and adverse events; and 5) Types of study: only RCTs published in a peer-reviewed journal were included.

Exclusion criteria filtered out studies were: 1) of non-RCT, animal studies, case reports, conference proceedings, or literature reviews; 2) with ambiguous diagnostics; 3) of incomplete data or unavailable full-text; or 4) of duplicates.

### Study selection and data extraction

Two independent reviewers (YX and JWW) extracted following data, such as the first author’s name, year of publication, study design, participants characteristics, specifics of CHF and control intervention, and outcomes metrics. Disputes were resolved by a third reviewer (YHC). All data underwent cross-checking before input into the RevMan software (V.5.3).

### Methodological quality assessment

Methodological quality of the included studies was rated by two reviewers independently (YX and YHC) with the Cochrane collaboration risk assessment tool. The risk of bias was evaluated across the following domains and classified as high, unclear, or low: 1) random sequence generation; 2) allocation concealment; 3) blinding of participants and personnel; 4) blinding of outcome assessors; 5) incomplete outcome data; 6) selective reporting; and 7) other bias. Any inconsistency was addressed by consulting a third reviewer (YHC).

### Data analysis

The quantitative synthesis was performed using RevMan software (v5.3). Risk ratio (RR) was used for dichotomous data and standard mean difference (SMD) or mean difference (MD) for continuous outcomes, each with 95% confidence intervals (CIs). Heterogeneity was measured using the Q-test and *I* ^2^ statistic, with a random-effects model applied for substantially heterogeneity (*I* ^2^ ≥ 50%) and a fixed-effects model otherwise. Publication bias was examined using funnel plots, and the robustness and reliability of the findings was tested with the sensitivity analysis by removing individual studies from the pooled data. A *P*-value less than 0.05 was considered statistically significant.

## Results

### Eligible studies

**FIGURE 1:**
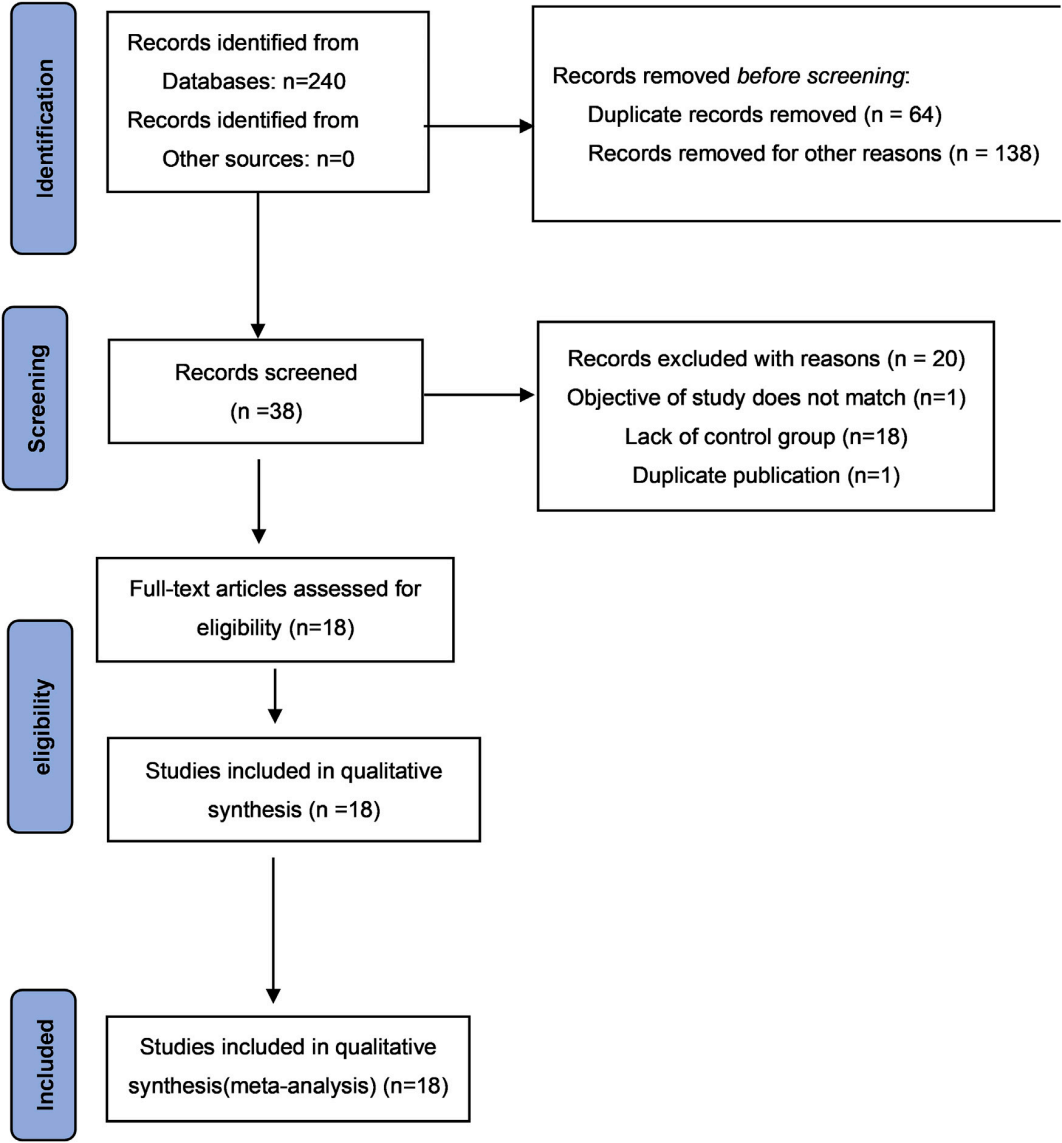
PRISMA flowchart for eligible study selection process.

Initially, 240 studies investigating CHF’s effectiveness and safety in dysmenorrhea treatment were retrieved. After eliminating 64 duplicated entries, the abstract and titles of remaining studies were screened to remove another 138 studies. A thorough review of the full text of the remaining 38 documents led to a further exclusion of 20 research due to the following reasons: one study with unrelated objective, 18 lack of control groups, and one duplication. Ultimately, 18 RCTs were included in the meta-analysis (Zhang, 2003; Qu, 2012; Qu and Li, 2012; Lei and Liu, 2013; Lei and Liu, 2014; Ye and Xing, 2017a; Ye and Xing, 2017b; Zhang, 2017; Liu et al., 2018; Yang, 2018; Yu and Lu, 2018; Yuan et al., 2018; Zhang, 2018; Zheng et al., 2019; Zheng and Li, 2019; Zheng, 2020a; Zheng, 2020b; Zheng, 2021). The PRISMA flowchart of the selection process is depicted in Figure 1.

### Study characteristics

This meta-analysis encompassed 18 RCTs with sample sizes ranging from 57 to 150 were fulfilled the pre-specified inclusion criteria, involving 1,484 dysmenorrhea patients. All trials were implemented in China and published in Chinese from 2003 to 2021. Dysmenorrhea type was distinctly identified in all studies, including primary dysmenorrhea in 12 studies (Zhang, 2003; Qu, 2012; Qu and Li, 2012; Ye and Xing, 2017a; Zhang, 2017; Liu et al., 2018; Yang, 2018; Yu and Lu, 2018; Yuan et al., 2018; Zhang, 2018; Zheng, 2020a; Zheng, 2021), secondary dysmenorrhea due to adenomyosis in four studies (Ye and Xing, 2017b; Zheng et al., 2019; Zheng and Li, 2019; Zheng, 2020b), and both primary and secondary dysmenorrhea in two studies (Lei and Liu, 2013; Lei and Liu, 2014). All control groups received oral medication, namely, Chinese patent medicine in four studies (Zhang, 2003; Yang, 2018; Zheng, 2020a; Zheng, 2021), Chinese herbal decoction in 13 studies (Qu, 2012; Qu and Li, 2012; Lei and Liu, 2013; Lei and Liu, 2014; Ye and Xing, 2017a; Ye and Xing, 2017b; Zhang, 2017; Liu et al., 2018; Yu and Lu, 2018; Yuan et al., 2018; Zhang, 2018; Zheng and Li, 2019; Zheng, 2020a), and conventional medicine (gestrinone) in one study (Zheng et al., 2019).

The patients in the trial groups were treated by CHF in combination with the same oral medications as the control group in 16 studies (Zhang, 2003; Qu, 2012; Qu and Li, 2012; Lei and Liu, 2013; Lei and Liu, 2014; Ye and Xing, 2017a; Ye and Xing, 2017b; Zhang, 2017; Liu et al., 2018; Yu and Lu, 2018; Yuan et al., 2018; Zhang, 2018; Zheng and Li, 2019; Zheng, 2020a; Zheng, 2020b; Zheng, 2021), with medication different from the control group in one study (Yang, 2018), and alone in one study (Zheng et al., 2019). Regarding outcome measurements, 15 studies reported the total effective rate (Zhang, 2003; Qu, 2012; Qu and Li, 2012; Lei and Liu, 2013; Lei and Liu, 2014; Ye and Xing, 2017a; Zhang, 2017; Liu et al., 2018; Yang, 2018; Yu and Lu, 2018; Yuan et al., 2018; Zhang, 2018; Zheng et al., 2019; Zheng, 2021), two studies exhibited the VAS (Zhang, 2018; Zheng et al., 2019), one study presented the CMSS (Zheng and Li, 2019), five studies noted symptom score (Lei and Liu, 2013; Yuan et al., 2018; Zhang, 2018; Zheng et al., 2019; Zheng, 2021), and four trials provided TCM syndrome scale (Qu, 2012; Zhang, 2018; Zheng, 2020a; Zheng, 2020b). The basic characteristics of the included trials are summarized in Table 2, the information of CHF formulas, preparation, and interventional details is presented in Table 3, and the detailed information of those highly-frequent used Chinese herbs (n > 5) is presented in Table 4.

**TABLE 2:** Characteristics of the included RCTs in this study.

Study ID	Arms	Type of dysmenorrhea	TCM pattern differentiation	Sample size	Average age(y)	Average course(y)	Intervention measures T./C		Outcome measures
				T./C	T./C	T./C	[Treatment duration (menstural cycle)/time/frequency/foot bathing temperature/depth]	Oral	
Zheng (2020a)	2	PD	qi stagnation and blood stasis	30/30	22.27 ± 5.66/21.53 ± 5.43	5.12 ± 3.88/4.55 ± 3.46	CHF (10d*3/30min/once per day/36°C–40°C/to ankle) +HJXJ capsule	HJXJ capsule	total effect rate, symptom score
Zheng (2021)	2	PD	qi stagnation and blood stasis	30/30	22.27 ± 5.66/21.53 ± 5.43	5.12 ± 3.88/4.55 ± 3.46	CHF (10d*3/30min/once per day/36°C/to ankle) + HJXJ capsule	HJXJ capsule	TCM syndrome scale, effective rate based on TCM syndrome, hemorrhrology
Zheng and Li (2019)	2	SD (adenomyosis)	yang deficiency and cold coagulation	54/54	31.51 ± 4.57/31.45 ± 5.01	5.32 ± 1.73/5.29 ± 1.75	CHF(7d*6/20min/once per day/40°C/foot) + ZYXZ decoction	ZYXZ decoction	total effective rate, TCM syndrome scale, CMSS
Yuan et al. (2018)	3	PD	qi stagnation and blood stasis	31/31/31	22.58 ± 3.25/23.46 ± 3.12/21.22 ± 3.08	7.86 ± 2.03/7.24 ± 2.55/7.88 ± 2.35	CHF (10d-15d*3/20min/once per day/NA/NA) + GXZY decoction	GXZY decoction	total effect rate, symptom score
Zhang (2018)	2	PD	cold coagulation and blood stasis	33/32	16–30	NA	CHF (10d*3/NR/qn/38°C/to the level of acupoint ST36) + LGDS decoction	LGDS decoction	total effect rate, effective rate based on TCM syndrome, symptom score, TCM syndrome scale, VAS
Ye and Xing (2017a)	2	SD (adenomyosis)	yang deficiency and cold coagulation	30/30	13–40	NA	CHF (8d*6/20min/once per day/40°C/foot) +ZYXZ decoction	ZYXZ decoction	total effect rate, CA125
Qu and Li (2012)	3	PD	cold coagulation and blood stasis	30/28/28	23.4/20.4/21.8	5.2/4.1/4.8	CHF (10d*3/15min/once per day/NA/foot) +SFZY decoction	SFZY decoction	total effect rate
Zhang (2003)	2	PD	NA	82/68	21.2/20.9	4.8/5.1	CHF (10d*3/15–20min/qg/NA/foot) + SFZY pill	SFZY pill	total effect rate
Zheng (2020b)	2	SD (adenomyosis)	yang deficiency and cold coagulation	29/29	31.18 ± 2.73	4.92 ± 1.64	CHF (14d*3/15–20min/once per day/35°C–40°C/to the ankle) + ZYXZ decoction	ZYXZ decoction	TCM syndrome scale
Zheng et al. (2019)	2	SD (adenomyosis)	cold coagulation and blood stasis	60/60	36.74 ± 8.51/36.39 ± 8.62	5.84 ± 1.70/5.76 ± 1.85	CHF (3/30min/once per day/40°C–50°C/NA) +WYSHZY decoction	gestrinone	total effect rate, symptom score, VAS
Yu and Lu (2018)	2	PD	Cold-damp coagulation	30/30	21.23 ± 2.84/20.97 ± 2.79	4.47 ± 1.57/4.34 ± 1.42	CHF (10d*3/30min/NR/40°C/to the ankle)	SFZY decoction	total effect rate, kupperman scale
Yang (2018)	2	PD	cold coagulation and blood stasis	39/39	19.8 ± 2.1/20.6 ± 1.4	56.3 ± 8.6/57.6 ± 8.3*	CHF (7d*3/10–15min/once per day/NA/foot) +TCM decoction	YueYue Shu granule	total effect rate, symptom score
Liu et al. (2018)	2	PD	cold coagulation and blood stasis	31/31	23.7 ± 3.5/24.0 ± 2.8	3.2 ± 1.3/3.4 ± 0.5	CHF (7d-10d*2/15min/once per day/38°C/10 cm above the ankle) + WJ decoction	WJ decoction	total effect rate
Ye and Xing. (2017b)	2	PD	NA	30/30	13–40	NA	CHF (10d-15d*3/20min/once per day/NA/foot) + ZYTJ decoction	ZYTJ decoction	total effect rate
Zhang (2017)	3	PD	cold coagulation and blood stasis	30/29/29	21.4 ± 2.76/21.53 ± 2.5/21.7 ± 2.51	5.32 ± 1.92/5.47 ± 1.94/4.87 ± 1.98	CHF (13d*3/30min/once per day/NA/to the level of acupoint SP6) + WJ decoction	WJ decoction	total effect rate, PGF2α
Lei and Liu (2013)	2	PD&SD	cold coagulation and blood stasis	30/30	26.4/26.8	2.3/2.5	CHF (10d-15d*3/20min/once per day/NA/foot) + WJ decoction	WJ decoction	total effect rate
Lei and Liu (2014)	2	PD&SD	cold coagulation and blood stasis	28/29	30.78 ± 2.94/30.20 ± 3.28	7.52 ± 5.92/7.11 ± 6.06	CHF (10d-15d*3/15–30min/once per day/NA/to ankle) + WJ decoction	WJ decoction	total effect rate, symptom score
Qu (2012)	3	PD	cold coagulation and blood stasis	36/35/34	22.61 ± 5.16/23.17 ± 4.69/22.81 ± 4.89	5.45 ± 4.35/5.86 ± 4.39/5.74 ± 4.06	CHF (10d*3/30min/once per day/35°C–40°C/to the ankle) + SFZY decoction	SFZY decoction	total effect rate, effective rate based on TCM syndrome, TCM syndrome scale, hemorrhrology

T., treatment group; C., control group; *month; NA, not available; PD, primary dysmenorrhea; SD, secondary dysmenorrhea; HJXJ, capsule, HongJin XiaoJie capsules; ZYXZ, decoction, ZhuYang XiaoZhen decoction; GXZY, decoction, GeXia ZhuYu decoction; LGDS, decocotion, LingGui DanShen decoction; ZYXZ, decoction, ZhuYang XiaoZhen decoction; SFZY, decoction, ShaoFu ZhuYu decoction; SFZY, pill, ShaoFu ZhuYu pill; WYSHZY, decoction, WenYang SanHan ZhuYu decoction; ZYTJ, decoction, ZhuYang TiaoJing decoction; WJ, decoction, WenJing decoction; CMSS, the Cox menstrual symptom scale; VAS, visual analogu sacle.

**TABLE 3:** Information of CHF formulas(g), intervention parameters, and TCM pattern differentiation.

Study ID	CHF components [Chinese name (family: Scientific name)] (dosage/g)	CHF parameters	TCM pattern Differentiation
Zheng (2020a)	Chai Hu [Bupleurum chinense DC., Bupleurum scorzonerifolium Willd.] 10g, Xiang Fu [Cyperus rotundus L.] 20g, Dang Gui [Angelica sinensis (Oliv.) Diels] 20g, Chuan Xiong [Ligusticum chuanxiong Hort.] 20g, Tao Ren [Prunus persica (L.) Batsch, Prunus davidiana (Carr.) Franch.] 10g, Hong Hua [Carthamus tinctorius L.] 10g, Yan Hu Suo [Corydalis yanhusuo W.T.Wang] 20g, Qing Pi [Citrus reticulata Blanco] 10g, Ji Xue Teng [Spatholobus suberectus Dunn] 20g, Yi Mu Cao [Leonurus japonicus Houtt.] 20 g	36°C–40°C/to the ankle/30min each time/7 days before menstruation, once a day for 10 consecutive days*3 menstrual cycle	qi stagnation and blood stasis
Zheng, 2021	Chai Hu [Bupleurum chinense DC., Bupleurum scorzonerifolium Willd.] 10g, Xiang Fu [Cyperus rotundus L.] 20 g, Tao Ren [Prunus persica (L.) Batsch, Prunus davidiana (Carr.) Franch.] 10g, Hong Hua [Carthamus tinctorius L.] 10g, Chuan Xiong [Ligusticum chuanxiong Hort.] 20g, Dang Gui [Angelica sinensis (Oliv.) Diels] 20g, Yan Hu Suo [Corydalis yanhusuo W.T.Wang] 20g, Qing Pi [Citrus reticulata Blanco] 10g, Yi Mu Cao [Leonurus japonicus Houtt.] 20g, Ji Xue Teng [Spatholobus suberectus Dunn] 20 g	36°C/to the ankle/30min each time/7 days before menstruation, once a day for 10 consecutive days*3 menstrual cycle	qi stagnation and blood stasis
Zheng and Li (2019)	Ai Ye [Artemisia argyi Levl.et Vant.] 30g, Niu Xi [Achyranthes bidentata Bl.] 30g, Ji Xue Teng [Spatholobus suberectus Dunn] 30g, Zhi Shou Wu [Polygonum multiflorum Thunb.] 20g, Gui Zhi [Cinnamomum cassia Presl] 20g, Chi Shao [Paeonia lactiflora Pall. and Paeonia veitchii Lynch] 20g, Dang Gui [Angelica sinensis (Oliv.) Diels] 15g, Shen Jin Cao [Lycopodium japonicum Thunb.] 15g, Hong Hua [Carthamus tinctorius L.] 10g, Chuan Xiong [Ligusticum chuanxiong Hort.] 10g, Cao Wu [Aconitum kusnezoffii Reichb.] 10g, Wu Zhu Yu [Euodia rutaecarpa (Juss.) Benth.] 10 g	40°C/foot/20min each time/7 days before and during menstruation, once a day for 6 menstrual cycles	yang deficiency and cold coagulation
Yuan et al. (2018)	Yi Mu Cao [Leonurus japonicus Houtt.]15g, Xiang Fu [Cyperus rotundus L.] 9g, Ai Ye [Artemisia argyi Levl.et Vant.] 15g, Hong Hua [Carthamus tinctorius L.] 9g, Yan Hu Suo [Corydalis yanhusuo W.T.Wang] 9 g	/NA/NA/20min each time/3–5 days before menstruation until the end of the period, once a day for 3 consecutive menstrual cycles	qi stagnation and blood stasis
Zhang (2018)	Ai Ye [Artemisia argyi Levl.et Vant.] 15g, Hua Jiao [Zanthoxylum schinifolium Sieb. et Zucc. and Zanthoxylum bungeanum Maxim.] 10g, Xiao Hui Xiang [Foeniculum vulgare Mill.] 15g, Niu Xi [Achyranthes bidentata Bl.] 10g, Yin Yang Huo [Epimedium brevicornu Maxim.] 20g, Hu Lu Ba [Trigonella foenum-graecum L.] 15 g	38°C/to the level of acupoint ST36/NR/qn/once a day before sleep for 10 consecutive days*3 menstrual cycles	cold coagulation and blood stasis
Ye and Xing (2017a)	Ai Ye [Artemisia argyi Levl.et Vant.] 30g, Zhi Shou Wu [Polygonum multiflorum Thunb.] 20g, Niu Xi [Achyranthes bidentata Bl.] 30g, Hong Hua [Carthamus tinctorius L.] 10g, Cao Wu [Aconitum kusnezoffii Reichb.] 10g, Dang Gui [Angelica sinensis (Oliv.) Diels] 15g, Chi Shao [Paeonia lactiflora Pall. And Paeonia veitchii Lynch] 20g, Chuan Xiong [Ligusticum chuanxiong Hort.] 10g, Gui Zhi [Cinnamomum cassia Presl] 20g, Shen Jin Cao [Lycopodium japonicum Thunb.] 15g, Ji Xue Teng [Spatholobus suberectus Dunn] 30g, Wu Zhu Yu [Euodia rutaecarpa (Juss.) Benth.] 10 g	40°C/foot/20 min each time/7 days before and during menstruation, once a day for 6 menstrual cycles	yang deficiency and cold coagulation
Qu and Li (2012)	Wu Zhu Yu [Euodia rutaecarpa (Juss.) Benth.]15g, Rou Gui [Cinnamomum cassia Presl]10g, Chuan Xiong [Ligusticum chuanxiong Hort.] 15g, Dang Gui [Angelica sinensis (Oliv.) Diels] 15g, Mu Dan Pi [Paeonia suffruticosa Andr.]15g, Xiang Fu [Cyperus rotundus L.] 15g, Shao Yao [Paeonia lactiflora Pall.] 15g, Xiao Hui Xiang [Foeniculum vulgare Mill.] 15g, Dan Shen [Salvia miltiorrhiza Bge.] 20g, Yan Hu Suo [Corydalis yanhusuo W.T.Wang] 15 g	NA/foot/15min each time/7 days before menstruation, once a day for 10 consecutive days	cold coagulation and blood stasis
Zhang (2003)	Dang Gui [Angelica sinensis (Oliv.) Diels]20g, Fu Zi [Aconitum carmichaelii Debx.] 15g, Xiao Hui Xiang [Foeniculum vulgare Mill.] 15g, Wu Zhu Yu [Euodia rutaecarpa (Juss.) Benth.] 15g, Chuan Jiao [Zanthoxylum schinifolium Sieb. et Zucc.] 10g, Xi Xin [Asarum heterotropoides Fr. Schmidt var. mandshuricum (Maxim.)Kitag.] 10g, Chai Hu [Bupleurum chinense DC., Bupleurum scorzonerifolium Willd.] 15g, Xiang Fu [Cyperus rotundus L.] 10g, Wu Ling Zhi [ ] 10g, Niu Xi [Achyranthes bidentata Bl.] 15g, Yan Hu Suo [Corydalis yanhusuo W.T.Wang] 15g, Ji Xue Teng [Spatholobus suberectus Dunn] 15 g	NA/foot/15–20min each time/qg/7 days before menstruation, once a day for 10 consecutive days	NA
Zheng (2020b)	Ai Ye [Artemisia argyi Levl.et Vant.] 30g, Ji Xue Teng [Spatholobus suberectus Dunn] 30g, Niu Xi [Achyranthes bidentata Bl.] 30g, Chi Shao [Paeonia lactiflora Pall. And Paeonia veitchii Lynch] 25g, Zhi Shou Wu [Polygonum multiflorum Thunb.] 25g, Gui Zhi [Cinnamomum cassia Presl]25g, Hong Hua [Carthamus tinctorius L.] 15, Dang Gui [Angelica sinensis (Oliv.) Diels] 15g, Cao Wu [Aconitum kusnezoffii Reichb.] 15g, Wu Zhu Yu [Euodia rutaecarpa (Juss.) Benth.] 15g, Shen Jin Cao [Lycopodium japonicum Thunb.] 15 g	35°C–40°C/to the ankle/15–20min each time/7 days before and after menstruation, once a day for 3 consecutive menstrual cycles	yang deficiency and cold coagulation
Zheng et al. (2019)	Dang Gui [Angelica sinensis (Oliv.) Diels] 15g, Wei Ling Xian [Clematis chinensis Osbeck] 15g, Gui Zhi [Cinnamomum cassia Presl]15g, Wu Zhu Yu [Euodia rutaecarpa (Juss.) Benth.] 10g, Chuan Xiong [Ligusticum chuanxiong Hort.] 10g, Dang Shen [Codonopsis pilosula (Franch.)Nannf.] 10g, Chi Shao [Paeonia lactiflora Pall. And Paeonia veitchii Lynch] 10g, Fa Ban Xia [Pinellia ernate (Thunb.) Breit.] 10g, Chai Hu [Bupleurum chinense DC., Bupleurum scorzonerifolium Willd.], Yan Hu Suo [Corydalis yanhusuo W.T.Wang] 10g, Hu Lu Ba [Trigonella foenum-graecum L.] 10g, Gan Cao [Glycyrrhiza uralensis Fisch.] 6 g	40°C–50°C/NA/30min each time/once a day for 3 menstrual cycles	cold coagulation and blood stasis
Yu and Lu (2018)	Dang Gui [Angelica sinensis (Oliv.) Diels]20g, Chuan Xiong [Ligusticum chuanxiong Hort.]10g, Gui Zhi [Cinnamomum cassia Presl]6g, Chi Shao [Paeonia lactiflora Pall. And Paeonia veitchii Lynch]10g, Pu Huang [Typha angustifolia L.] 10g, Wu Ling Zhi [ ]10g, Mo Yao [Commiphora myrrha Engl.] 10g, Yan Hu Suo [Corydalis yanhusuo W.T.Wang] 20g, Gan Jiang [Zingiber officinale Rosc.] 6g, Xiao Hui Xiang [Foeniculum vulgare Mill.] 6 g. Modification: add Ai Ye [Artemisia argyi Levl.et Vant.] 10g, Wu Zhu Yu [Euodia rutaecarpa (Juss.) Benth.]15 g for sever cold pain; add Xiang Fu [Cyperus rotundus L.] 15g, Wu Yao [Lindera ggregate (Sims) Kos-term.] 15 g for severe abdominal bloating	40°C/to the ankle/30min each time/NR/once a day for 10 days * 3 menstrual cycles	Cold-damp coagulation
Yang (2018)	Gui Zhi [Cinnamomum cassia Presl] 10g, Lu Lu Tong [Liquidambar formosana Hance] 10g, Yin Yang Huo [Epimedium brevicornu Maxim.]10g, Zhi Chuan Wu [Aconitum carmichaelii Debx.] 9g, Zhi Cao Wu [Aconitum kusnezoffii Reichb.] 9g, Wu Zhu Yu [Euodia rutaecarpa (Juss.) Benth.] 6g, Chuan Xiong [Ligusticum chuanxiong Hort.]6g, Xi Xin [Asarum heterotropoides Fr. Schmidt var. mandshuricum (Maxim.)Kitag.]4 g	NA/foot/10–15min each time/once a day/7 days before menstruation and stop using when period arrives, once a day for 3 menstrual cycles	cold coagulation and blood stasis
Liu et al. (2018)	Yi Mu Cao [Leonurus japonicus Houtt.]30g, Xiao Hui Xiang [Foeniculum vulgare Mill.]15g, Yan Hu Suo [Corydalis yanhusuo W.T.Wang] 15g, Niu Xi [Achyranthes bidentata Bl.] 15g, Chao Pu Huang [Typha angustifolia L.] 15g, Ji Xue Teng [Spatholobus suberectus Dunn] 15g, Hua Jiao [Zanthoxylum schinifolium Sieb. et Zucc. and Zanthoxylum bungeanum Maxim.] 10g, Wu Zhu Yu [Euodia rutaecarpa (Juss.) Benth.] 10g, Chuan Xiong [Ligusticum chuanxiong Hort.] 10 g	38°C/10 cm above the ankle/15min each time/7–10 consecutive days before menstruation until the end of period, once a day for 2 consecutive menstrual cycles	cold coagulation and blood stasis
Ye and Xing (2017b)	before menstruation: Tu Si Zi [Cuscuta australis R.Br. and Cuscuta chinensis Lam.], Xu Duan [Dipsacus asper Wall. Ex Henry], Dan Shen [Salvia miltiorrhiza Bge.], Chi Shao [Paeonia lactiflora Pall. and Paeonia veitchii Lynch], Shao Yao [Paeonia lactiflora Pall.], Shan Zhu Yu [Cornus officinalis Sieb. et Zucc.], Mu Dan Pi [Paeonia suffruticosa Andr.], Fu Ling [Poria cocos (Schw.) Wolf], Zi Shi Ying [Fluoritum], Mu Xiang [Aucklandia lappa Decne.], Chai Hu [Bupleurum chinense DC., Bupleurum scorzonerifolium Willd.]; during menstruation: Dang Gui [Angelica sinensis (Oliv.) Diels], Chi Shao [Paeonia lactiflora Pall. and Paeonia veitchii Lynch], Chuan Xiong [Ligusticum chuanxiong Hort.], E Zhu [Curcuma phaeocaulis VaL.], Xiang Fu [Cyperus rotundus L.], Mei Gui Hua [Rosa rugosa Thunb.], Yi Mu Cao [Leonurus japonicus Houtt.], Chuan Niu Xi [Achyranthes bidentata Bl.], Tao Ren [Prunus persica (L.) Batsch, Prunus davidiana (Carr.) Franch.], Ji Xue Teng [Spatholobus suberectus Dunn], Rou Gui [Cinnamomum cassia Presl], Yan Hu Suo [Corydalis yanhusuo W.T.Wang]; Modification: add Du Zhong [Eucommia ulmoides Oliv.] for severe sore lower back, Wu Zhu Yu [Euodia rutaecarpa (Juss.) Benth.] for cold pain in the lower abdomen, Pu Huang [Typha angustifolia L.] and Wu Ling Zhi [Trogopterus xanthippes Milne-Edwards] for severe menstrual clots and blood stasis, and Gan Jiang [Zingiber officinale Rosc.] for nausea and vomiting	NA/foot/20min each time/once a day/3–5days before menstruation until the end of period, once a day for 3 consecutive menstrual cycles	NA
Zhang (2017)	Rou Gui [Cinnamomum cassia Presl]20g, Xiao Hui Xiang [Foeniculum vulgare Mill.]20g, Dang Gui [Angelica sinensis (Oliv.) Diels]15g, Chuan Xiong [Ligusticum chuanxiong Hort.] 15g, Niu Xi [Achyranthes bidentata Bl.] 15g, Yan Hu Suo [Corydalis yanhusuo W.T.Wang] 30g, Dan Shen [Salvia miltiorrhiza Bge.] 15g, Chi Shao [Paeonia lactiflora Pall. and Paeonia veitchii Lynch] 20g, Bai Shao [Paeonia lactiflora Pall.] 20 g	NA/to the level of acupoint SP6/30min each time/7 days before menstruation till the third day of menstruation, once a day for 3 consecutive menstrual cycles	cold coagulation and blood stasis
Lei and Liu (2013)	Wu Zhu Yu [Euodia rutaecarpa (Juss.) Benth.] 10g, Dang Gui [Angelica sinensis (Oliv.) Diels] 15g, Chuan Xiong [Ligusticum chuanxiong Hort.]10g, Bai Shao [Paeonia lactiflora Pall.] 15g, Fa Ban Xia [Pinellia ernate (Thunb.) Breit.] 15g, Mai Men Dong [Ophiopogon japonicus (L.f) Ker-Gawl.] 10g, Dang Shen [Codonopsis pilosula (Franch.)Nannf.] 15g, Mu Dan Pi [Paeonia suffruticosa Andr.] 10g, Gui Zhi [Cinnamomum cassia Presl] 15g, Gan Jiang [Zingiber officinale Rosc.] 10g, Gan Cao [Glycyrrhiza uralensis Fisch.] 6 g	NA/to the ankle/about 20 min each time/3–5days before menstruation till the end of the period, once a day for 3 consecutive menstrual cycles	cold coagulation and blood stasis
Lei and Liu (2014)	Wu Zhu Yu [Euodia rutaecarpa (Juss.) Benth.] 10g, Dang Gui [Angelica sinensis (Oliv.) Diels] 15g, Chuan Xiong [Ligusticum chuanxiong Hort.]10g, Bai Shao [Paeonia lactiflora Pall.] 15g, Fa Ban Xia [Pinellia ternata (Thunb.) Breit.] 15g, Mai Men Dong [Ophiopogon japonicus (L.f) Ker-Gawl.] 10g, Dang Shen [Codonopsis pilosula (Franch.)Nannf.] 15g, Mu Dan Pi [Paeonia suffruticosa Andr.] 10g, Gui Zhi [Cinnamomum cassia Presl] 15g, Gan Jiang [Zingiber officinale Rosc.] 10g, Gan Cao [Glycyrrhiza uralensis Fisch.] 6 g	NA/foot/15–30min each time/3–5days before menstruation till the end of the period, once a day for 3 consecutive menstrual cycles	cold coagulation and blood stasis
Qu (2012)	Wu Zhu Yu [Euodia rutaecarpa (Juss.) Benth.]15g, Rou Gui [Cinnamomum cassia Presl] 15g, Dang Gui [Angelica sinensis (Oliv.) Diels] 15g, Chuan Xiong [Ligusticum chuanxiong Hort.] 10g, Bai Shao [Paeonia lactiflora Pall.] 30g, Mu Dan Pi [Paeonia suffruticosa Andr.] 10g, Gan Jiang [Zingiber officinale Rosc.] 10g, Yan Hu Suo [Corydalis yanhusuo W.T.Wang] 10g, Xiang Fu [Cyperus rotundus L.] 10g, Wu Yao [Lindera aggregata (Sims) Kos-term.] 10g, Gan Cao [Glycyrrhiza uralensis Fisch.] 10 g	35°C–40°C/to the ankle/30min each time/3–5days before menstruation till the end of the period, once a day for 10 consecutive days*3 menstrual cycles	cold coagulation and blood stasis

**TABLE 4:** Information of Chinese herbs that highly-frequent used to relieve dysmenorrhea in the 18 CHF prescriptions included by this study (n ≥ 5 times).

No.	Herbal name	Scientific name	TCM Category/Sub-Category	TCM	TCM property& flavor	Pharmacological effects	Frequency
				Function			
1	Chuanxiong Rhizoma (Chuan Xiong, 川芎)	Ligusticum chuanxiong Hort	Blood invigorating and stasis-dissolving/Blood-invigorating and pain-relieving	Activate blood and regulate qi, disperse wind and arrest pain	Warm; Pungent	Anti-myocardial ischemia, anti-cerebral ischemia, vasodilation, antiplatelet aggregation, antithrombosis, microcirculation improvement, antihyperlipidemic, cardiac regulation, stimulatory effect on uterine smooth muscle, sedative, improving immune and hematopoietic functions, antineoplastic, and analgesic effects ( Chen et al., 2018a; Li et al., 2020; Zhang et al., 2020; Liu et al., 2022 )	14
	6–20 g						
1′	Angelicae Sinensis Radix (Dang Gui, 当归)	Angelica sinensis (Oliv.) Diels	Deficiency-supplementing/Blood-supplementing	Tonify and activate blood, regulate menstruation and arrest pain, moisten the intestines and promote defecation	Warm; sweet, pungent	Antianemic, menstrual-pain arresting, anti-inflammatory, analgesic, antioxidant, antihyperlipidemic, anti-atherosclerosis, anti-myocardial ischemia, antiarrhythmic, protecting cardiomyocytes, vasodilation, antihypertensive, and anti-radiation effects; promoting bone marrow hematopoietic functions, inhibition of platelet aggregation, antithrombosis, improving hemorrheology, regulating uterine smooth muscle, enhancing immune functions, and hepatoprotection ( Li et al., 2022; Li et al., 2023b)	14
	15–20 g						
2	Euodiae Fructus (Wu Zhu Yu, 吴茱萸)	Euodia rutaecarpa (Juss.) Benth., Euodia rutaecarpa (Juss.) Benth. var. officinalis (Dode) Huang, Euodia rutaecarpa (Juss.) Benth. var. bodinieri (Dode) Huang	Interior-warming	Disperse cold and arrest pain, direct counterflow downward and arrest vomiting, assist yang and arrest diarrhea	Warm; bitter, pungent	Anticancer, antibacterial, anti-inflammatory, analgesic, antinociceptive, vasoconstrictive and vasodilator, anti-platelet, anti-arrhythmia, neuroprotective, anti-obesity and anti-diabetic, hepatorenal protection, insecticide, and anti-diarrheal effect ( Huang et al., 2016 )	13
	6–15 g						
3	Corydalis Rhizoma (Yan Hu Suo, 延胡索)	Corydalis yanhusuo W.T.Wang	Blood-invigorating and stasis-dissolving/Blood-invigorating and pain-relieving	Activate blood, regulate qi, and arrest pain	Warm; bitter, pungent	Analgesic, sedative, hypnosis, anti-myocardial ischemia, anti-cerebral ischemia, antineoplastic, and anti-ulcer effects; inhibition of platelet aggregation, spasmolysis, inhibiting gastric acid secretion, mediating endocrine system effects (Liu et al., 2021b; Kong et al., 2020; Wang et al., 2022a; Wang et al., 2023a; Wang et al., 2023c)	11
	9–30 g						
4′	Paeoniae Radix Rubra (Chi Shao, 赤芍)	Paeonia lactiflora Pall	Heat-clearing/Heat-clearing and blood-cooling	Clear heat, cool blood, dissolve stasis, and arrest pain	Mild cold; bitter	Hepaprotective, anti-inflammatory, anti-oxidative, anti-cardiovascular, microcirculation-improvement, blood vessels dilating, anti-myocardial ischemia, and anti-thrombosis activities ( Ke et al., 2017; Tan et al., 2020; Han et al., 2023; Gao et al., 2024; Sun et al., 2024 )	8
	10–25 g	Paeonia veitchii Lynch					
4′	Cinnamomi Ramulus (Gui Zhi, 桂枝)	Cinnamomum cassia (L.) J.Pres	Exterior-releasing/Exterior wind-cold dispersing	Induce sweating, release the flesh, warm and unblock the channels, assist yang and transform qi, calm surging and direct counterflow downward	Warm; sweet, pungent	Antitumor, anti-inflammation, analgesic, antidiabetic, anti-obesity, antibacterial, antiviral, cardiovascular protective, cytoprotective, neuroprotective, immunoregulatory, and anti-tyrosinase activities, vasodilation, diaphoretic, anti-pathogenic microorganism, improving cardiovascular functions, antipyretic, analgesic, anti-inflammatory, antiallergic, sedative, anti-convulsion, diuretic, antineoplastic, promoting peristalsis, antiplatelet aggregation, and cholagogic effects ( Liu et al., 2020; Liu et al., 2020; Zhang et al., 2021; Chen et al., 2023 ; Dang et al., 2020; Dang et al., 2023; Jo et al., 2023; Zhao et al., 2023; Li et al., 2024b; Ma et al., 2024 )	8
	6–25 g	Cinnamomum cassia Presl					
4″	Spatholobi Caulis (Ji Xue Teng, 鸡血藤)	Spatholobus suberectus Dunn	Blood-invigorating and stasis-dissolving/Blood-invigorating and menstruation-regulating	Activate and supplement blood, regulate menstruation and arrest pain, relax the sinews and quicken the collaterals	Warm; bitter, sweet	Anti-tumor, haematopoietic, anti-inflammatory, antidiabetic, antioxidant, antiviral, antibacterial effects; nervous system-regulating, antiviral, anti-osteoclastogenic, antidepressant and hepatoprotective effects ( Bae et al., 2022; Huang et al., 2023; Pan et al., 2023; Chen et al., 2024a )	8
	15–30 g						
5	Achyranthis	Achyranthes bidentata Bl	Blood-invigorating and stasis-dissolving/Blood-invigorating and menstruation-regulating	expel stasis and unblock menstruation, tonify the liver and kidney, strengthen sinews and bones, promote urination and relieve strangury	Neutral; bitter, sweet, sour	Anti-tumor, anti-inflammatory, anti-osteoporosis, and anti-atherosclerosis effects; regulating immune system, hypoglycemic, and lowering blood lipids ( Wang et al., 2020; Yang et al., 2020; An et al., 2023; Chai et al., 2024 )	7
	Bidentatae Radix (Niuxi, 怀牛膝)						
	10–30 g						
5′	Cyperi Rhizoma (Xiang Fu, 香附)	Cyperus rotundus L	Qi-regulating	soothe the liver and resolve constraint, regulate qi and loosen the center, regulate menstruation and arrest pain	Neutral; pungent, mild bitter, mild sweet	Analgesic, anti-allergic, anti-arthritic,anticariogenic, anticonvulsant, antidiarrheal, antiemetic, antihyperglycemic, antihypertensive, anti-inflammatory, antimalarial, anti-obesity, antioxidant, antiplatelet, antipyretic, anti-ulcer, antiviral, cardioprotective, gastroprotective, hepatoprotective, neuroprotective, ovicidal, larvicidal, relaxing intestinal muscle, inhibiting uterine smooth muscle, estrogen-like effect, antipyretic, anti-inflammatory, and analgesic effects (Jia et al., 2019; Kamala et al., 2018; Chen et al., 2022a; Wang et al., 2022b )	7
	9–20 g						
6	Artemisiae	Artemisia argyi Levl.et Vant	Bleeding-arresting/Channel-warming and bleeding-arresting	Warm the channels and arrest bleeding, disperse cold and arrest pain	Warm; bitter, pungent	Antibacterial, antiviral, hemostatic, anti-tumor, antioxidant, analgesic and anti-inflammatory effects; hepatoprotection, cough relief and asthma alleviation, blood sugar reduction, and immune regulation ( Ekiert et al., 2020; Lan et al., 2020; Han et al., 2022; Su et al., 2022 )	6
	Argyi Folium (Ai Ye, 艾叶)						
	10–30 g						
6′	Paeoniae Radix Alba (Bai Shao, 白芍)	Paeonia lactiflora Pall	Deficiency-supplementing/Blood-supplementing	Nourish the blood and regulate menstruation, restrain yin and arrest sweating, soften the liver and arrest pain, calm and inhibit liver yang	Mild cold; bitter, sour	Anti-inflammatory, antioxidant, antithrombotic, anticonvulsant, analgesic, cardioprotective, neuroprotective, hepatoprotective, antidepressant-like, antitumor, and immunoregulatory effects (Li et al., 2020; Chen et al., 2022b; Zhao et al., 2022a; Zhao et al., 2022b)	6
	15–30 g						
6″	Carthami Flos (Hong Hua,红花)	Carthamus tinctorius L	Blood-invigorating and stasis-dissolving/Blood-invigorating and menstruation-regulating	Activate blood and unblock menstruation, relieve blood stasis and arrest pain	Warm; pungent	Anti-thrombosis, anticoagulant, vasodilative, anti-atherosclerosis, anti-inflammatory, antioxidant, anti-depression, cardioprotective, cerebrovascular-protective, neuroprotective, hepatoprotctive, anti-tumor, anti-aging, anti-obesity; anti-inflammatory, and analgesic effects; lowering blood pressure improving hemorheology and myocardial ischemia, regulating lipid metabolism, immune, and gastrointestinal motility, and improving glucose metabolism and skin microcirculation ( Liang and Wang, 2022; Li et al., 2023c; Yuan et al., 2023; Bai et al., 2024; Yang et al., 2024 )	6
	9–15 g						
6‴	Foeniculi Fructus (Xiao Huixiang, 小茴香)	Foeniculum vulgare Mill	Interior-warming	Dissipate cold and arrest pain, regulate qi and harmonize the stomach	Warm; pungent	Anti-inflammatory, antipyretic, anti-anxiety, hepatorena-protective, anti-hepatic fibrosis, anti-oxidant, anti-stress, anti-aging, anti-bacterial, anti-viral, anti-tumor, anti-parasitic, neuroprotective, and analgesic effects; regulating gastrointestinal function, improving cognitive functions, lowering blood lipids and blood sugar, regulating estrogen levels, and enhancing immunity ( Wang et al., 2020; Choi et al., 2023 )	6
	6–20 g						
7	Bupleuri Radix (Chai Hu, 柴胡)	Bupleurum chinense DC	Exterior-releasing/Wind-heat dispersing	Scatter and dissipate external wind and heat退热, soothe the liver and resolve constraint, raise and lift yang qi	Mild cold; bitter, pungent	antipyretic, anti-inflammatory, anti-pathogenic microorganism, anti-bechic, anti-epileptic, hepatoprotective, cholagogic, anti-bacterial endotoxin, antihyperlipidemic, antidepressive, antineoplastic, sedative, and analgesic effects; regulating visceral smooth muscle, regulating protein, glucose and lipid metabolism, and improving immune functions (Zhao et al., 2022a; Tran et al., 2023; Chen et al., 2024b)	5
	10–15 g	Bupleurum scorzonerifolium Willd					
7′	Zingiberis Rhizoma (Gan Jiang, 干姜)	Zingiber officinale Rosc	Interior-warming	warm the center and dissipate cold, restore yang and unblock the channels, warm the lung and dissolve rheum (fluid retention)	Hot; pungent	Antiemetic, antibacterial, antitumor, anti-ulcer, antioxidant, anti-inflammatory, anti-stress, antipyretic, antithrombosis, antiallergic, antibechic, antioxidant, sedative, cholagogic, hepatoprotective, and analgesic effects; regulating gastrointestinal smooth muscle, cardiotonic, regulating blood vessel and pressure, inhibiting platelet aggregation, and improving immune functions ( Dang et al., 2020; Lai et al., 2022; Fang et al., 2024 )	5
	6–10 g						
7″	Moutan Cortex (Mu Danpi, 牡丹皮)	Paeonia suffruticosa Andr	Heat-clearing/Heat-clearing and blood-cooling	heat-clearing and blood-cooling invigorate blood and dissolve stasis	Mild cold; bitter, pungent	Antioxidant, anti-inflammatory, anti-oxidant, anti-tumor, and analgesic effects; hepato- and renal- protection, regulating metabolism, protecting nervous system, lowering blood sugar and blood pressure, and regulating blood lipids ( Cheng et al., 2018b; Liu et al., 2023; Wang et al., 2023b )	5
	10–15 g						
7‴	Leonuri Herba (Yi Mucao, 益母草)	Leonurus japonicus Houtt	Blood-invigorating and stasis-dissolving/Blood-invigorating and menstruation-regulating	Invigorate blood and regulate menstruation, promote urination and relieve edema, clear heat and resolve toxins	Mild cold; Bitter, pungent	Anti-thrombosis, anti-prostatic hyperplasia; improving hemorheology, microcirculation, myocardial ischemia, myocardial antioxidant capacity; stimulating effect on uterine smooth muscle, diuretic, preventing and treating acute renal tubular necrosis, and enhancing immune function ( Zhang et al., 2015; Wu et al., 2023a )	5
	15–30 g						

### Risk of bias assessment

**FIGURE 2:**
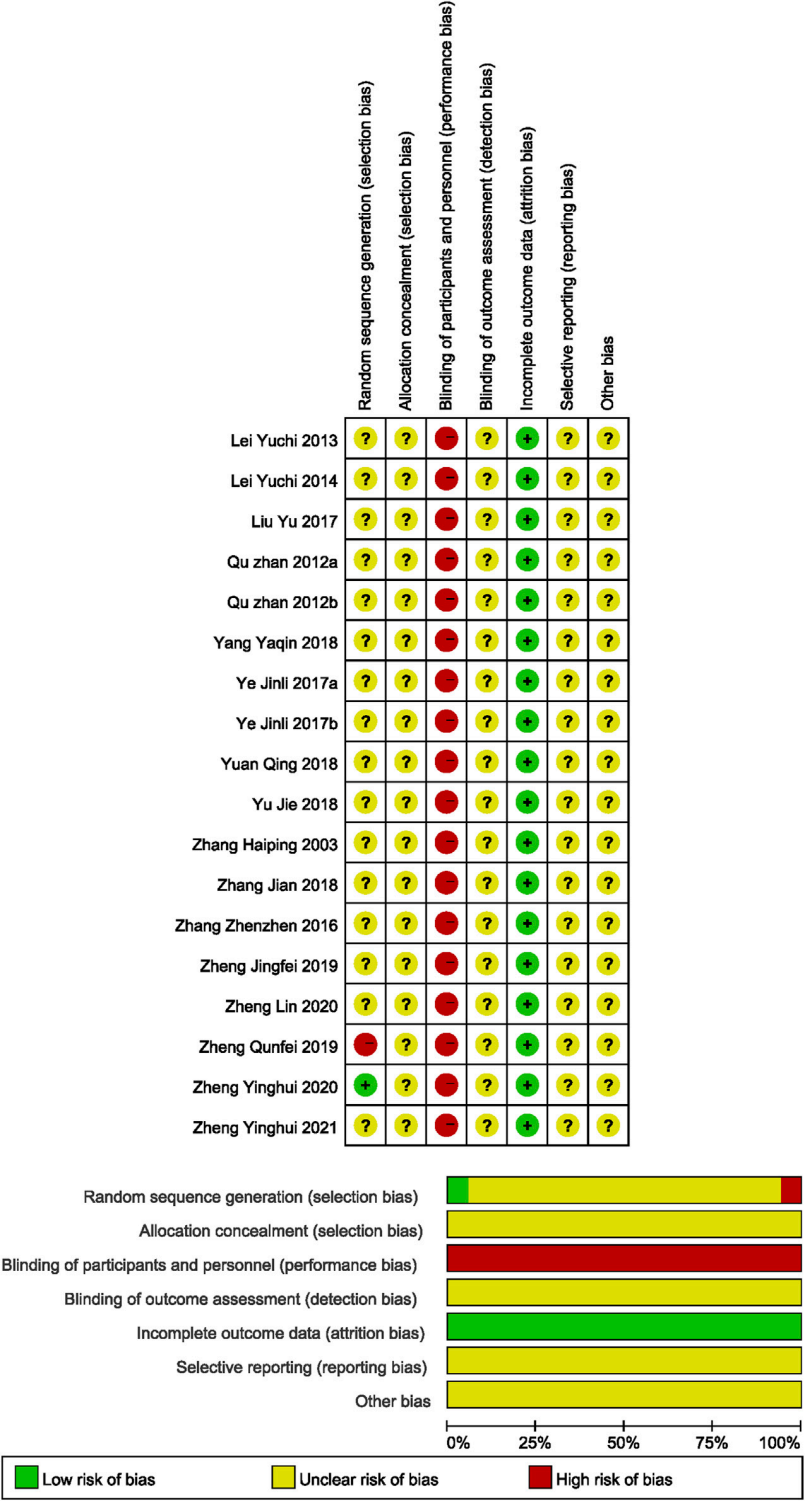
Assessment of methodological quality by the Cochrane risk-of-bias tool.

As shown in Figure 2, the methodological quality of the included studies was relatively low. All included studies claimed to be randomized, and one described the randomization method (Zheng, 2020a). Due to the inherent nature of the interventions, participant blinding was unfeasible in these studies, and none of them clarified their blinding procedures. All the studies mentioned but did not detail the process of allocation concealment or outcome assessment. Each study presented complete data. The risks of selective reporting and other biases were remained unclear due to insufficient information. The detailed results are presented in Figure 2.

### Effectiveness and safety of CHF therapy

#### Total effective rate

**FIGURE 3:**
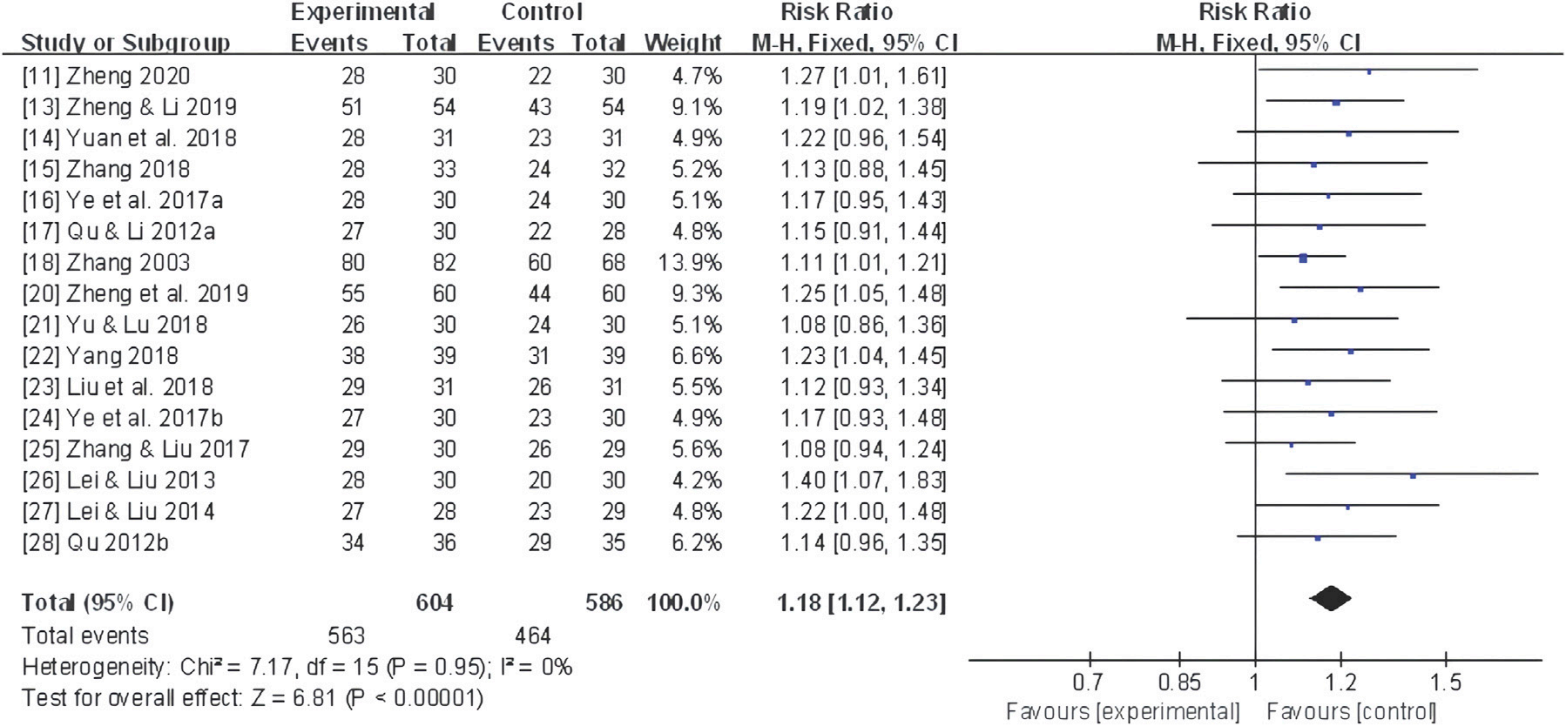
Forest plot for the total effective rate of adjunctive CHF therapy *versus* control group. CHF, Chinese herbal footbaths.

Sixteen studies (Zhang, 2003; Qu, 2012; Qu and Li, 2012; Lei and Liu, 2013; Lei and Liu, 2014; Ye and Xing, 2017a; Ye and Xing, 2017b; Zhang, 2017; Liu et al., 2018; Yang, 2018; Yu and Lu, 2018; Yuan et al., 2018; Zhang, 2018; Zheng et al., 2019; Zheng and Li, 2019; Zheng, 2020b) reported the total effective rate, and a fixed-effect model was utilized due to mild heterogeneity across studies (*I* ^*2*^ = 0%). The meta-analysis of the pooled data demonstrated that CHF as an adjuvant therapy yielded a statistically significant improvement in the total effective rate (RR 1.18, 95% CI: 1.12 to 1.23, *P* < 0.00001) (Figure 3).

**FIGURE 4:**
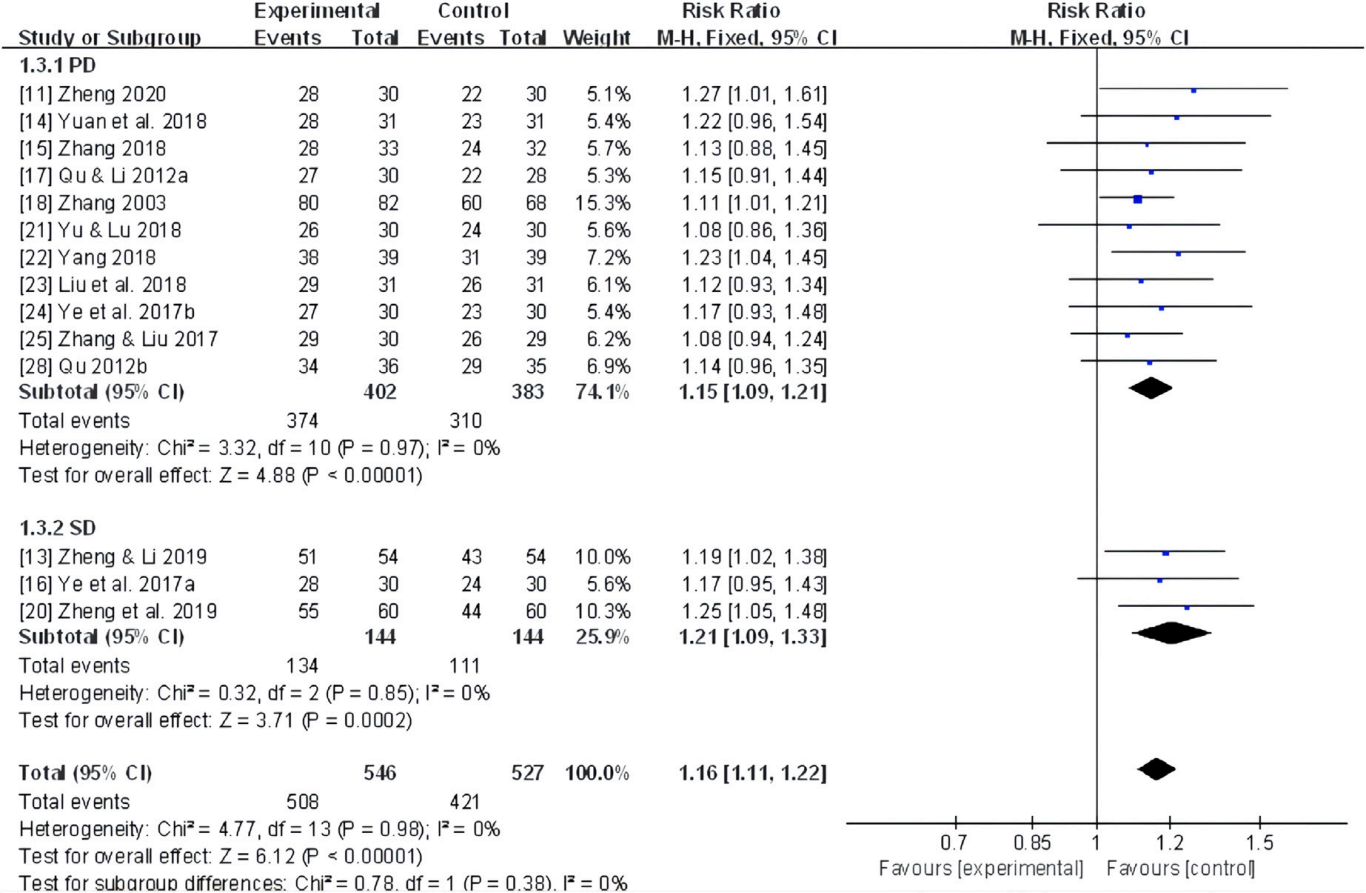
Forest plot for the total effective rate of adjunctive CHF therapy *versus* control group in terms of PD and SD. PD, Primary dysmenorrhea. SD, Secondary dysmenorrhea.

**FIGURE 5:**
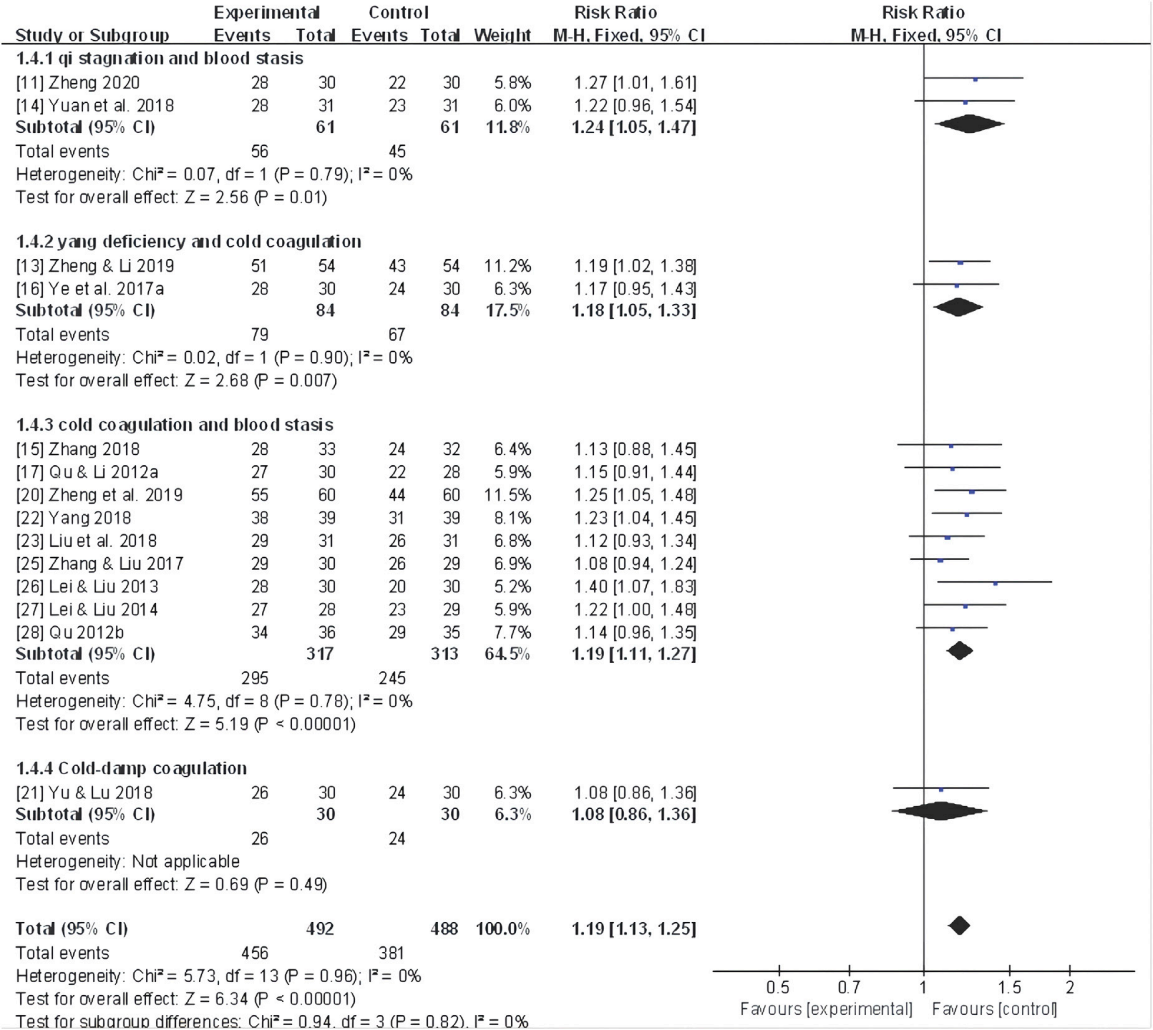
Forest plot for the total effective rate of adjunctive CHF therapy *versus* control group in terms of different TCM Patterns.

Further subgroup analyses were conducted based on different dysmenorrhea types and TCM patterns, revealing a significant improvement in the total effective rates in 11 trials (Zhang, 2003; Qu, 2012; Qu and Li, 2012; Ye and Xing, 2017b; Zhang, 2017; Liu et al., 2018; Yang, 2018; Yu and Lu, 2018; Yuan et al., 2018; Zhang, 2018; Zheng, 2020a) of primary dysmenorrhea (RR 1.15, 95% CI: 1.09 to 1.21, *P* < 0.00001), three trials (Ye and Xing, 2017a; Zheng et al., 2019; Zheng and Li, 2019) of secondary dysmenorrhea (RR 1.21, 95% CI: 1.09 to 1.33, *P* < 0.00001) (Figure 4), as well as two trials (Yuan et al., 2018; Zheng, 2020b) of TCM patterns of qi stagnation and blood stasis (RR 1.24, 95% CI: 1.05 to 1.47, *P* < 0.01), two trials (Ye and Xing, 2017b; Zheng and Li, 2019) of yang deficiency and cold coagulation (RR 1.18, 95% CI: 1.05 to 1.33, *P* < 0.01), and eight trials (Qu, 2012; Qu and Li, 2012; Lei and Liu, 2013; Lei and Liu, 2014; Zhang, 2017; Liu et al., 2018; Yang, 2018; Zhang, 2018; Zheng et al., 2019) of cold coagulation and blood stasis (RR 1.19, 95% CI: 1.11 to 1.27, *P* < 0.00001). A moderate improvement was also noted for cold-dampness coagulation pattern, but without statistical significance (RR 1.08, 95% CI: 0.86 to 1.36, *P* = 0.49, *I* ^*2*^ = Not applicable) (Figure 5).

#### VAS and CMSS

Two studies (Zhang, 2018; Zheng et al., 2019) reported the VAS, and a fixed-effect model was adopted due to the mild heterogeneity (*I* ^*2*^ = 1%). The aggravated effect of meta-analysis showed that CHF adjunctive intervention led to a significant decline in the VAS (MD 0.88, 95% CI: 0.68 to 1.09, *P* < 0.00001) (Supplementary Figure S1). Another study (Zheng and Li, 2019) reported a substantial reduction in the CMSS (MD 3.61, 95% CI: 2.73 to 4.49, *P* < 0.00001) in the CHF trial group as compared to the control group (Supplementary Figure S2).

#### Symptom score

**FIGURE 6:**
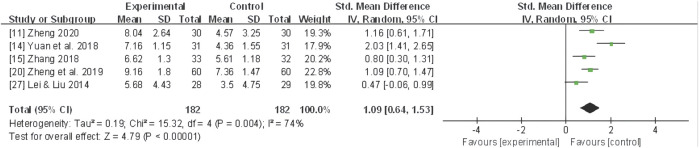
Forest plot for symptom score of adjunctive CHF therapy *versus* control group.

Five studies (Lei and Liu, 2014; Yuan et al., 2018; Zhang, 2018; Zheng et al., 2019; Zheng, 2020a) assessed the symptom score, and a random-effect model was applied due to the significant heterogeneity (*I* ^*2*^ = 74%). The meta-analysis of pooled data demonstrated that compared to the control group, CHF as an adjunctive intervention markedly reduced the symptom score (SMD 1.09, 95% CI: 0.64 to 1.53, *P* < 0.00001) (Figure 6).

**FIGURE 7:**
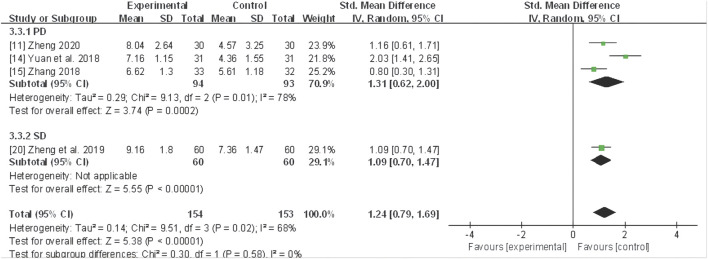
Forest plot for symptom score of CHF subgroup analysis on primary and secondary dysmenorrhea. CHF, Chinese herbal footbaths.

Further subgroup analyses based on different dysmenorrhea types demonstrated that a significant reduction in the symptom score in three trials (Yuan et al., 2018; Zhang, 2018; Zheng, 2020b) with primary dysmenorrhea (SMD 1.31, 95% CI: 0.62 to 2.00, *P*< 0.001) and one trial (Zheng et al., 2019) with secondary dysmenorrhea due to adenomyopathy (SMD 1.09, 95% CI: 0.70 to 1.47, *P* < 0.00001) (Figure 7).

#### TCM syndrome scale

**FIGURE 8:**
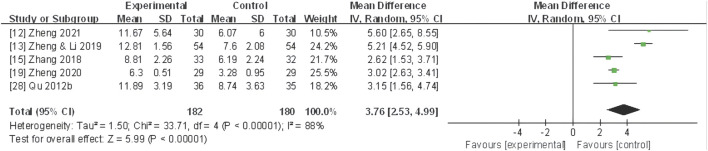
Forest plot for TCM syndrome scale of adjunctive CHF therapy *versus* control group. TCM, traditional Chinese medicine; CHF, Chinese herbal footbaths.

Five studies (Qu, 2012; Zhang, 2018; Zheng and Li, 2019; Zheng, 2020a; Zheng, 2021) evaluated the TCM syndrome scale, and a random-effect model was utilized due to pronounced heterogeneity (*I* ^*2*^ = 88%). The meta-analysis unveiled that CHF application substantially improved the TCM syndrome scale compared to the control group (MD 3.76, 95% CI: 2.53 to 4.99, *P* < 0.0001) (Figure 8).

**FIGURE 9:**
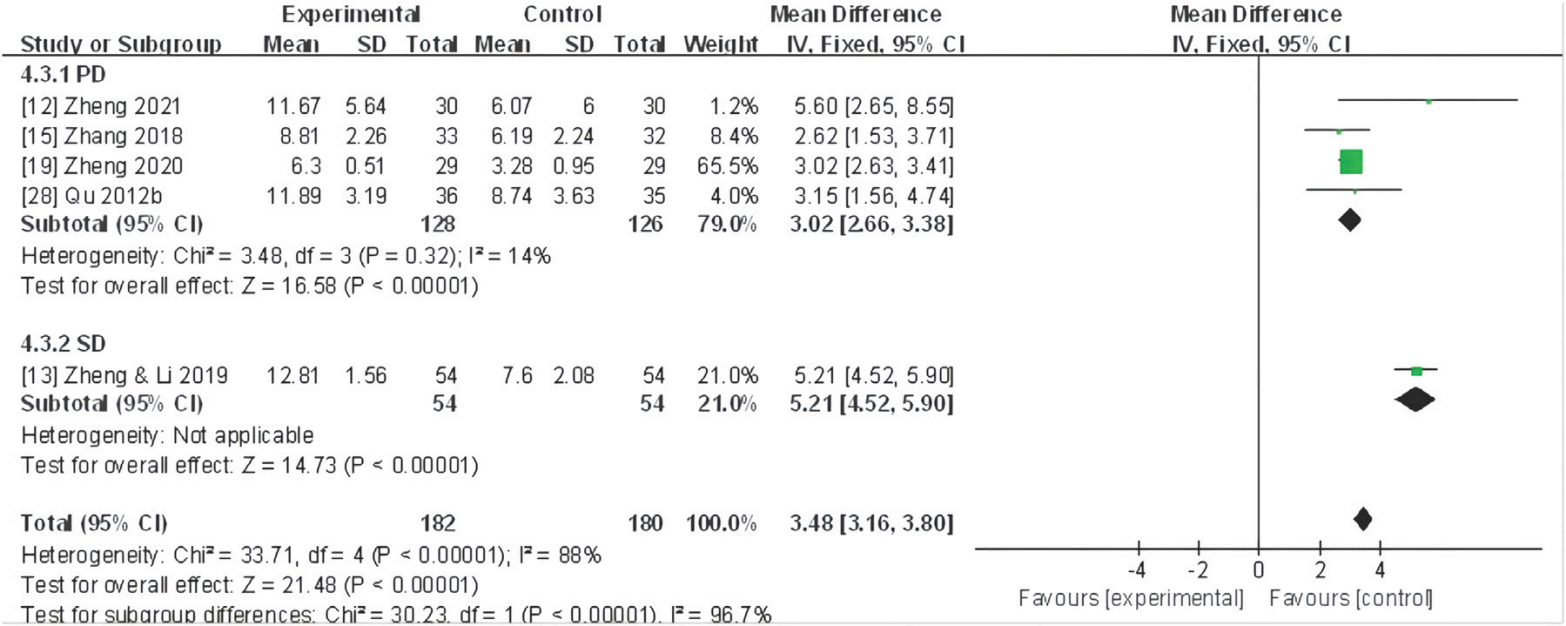
Forest plot for TCM syndrome scale of CHF subgroup analysis on primary and secondary dysmenorrhea.

**FIGURE 10:**
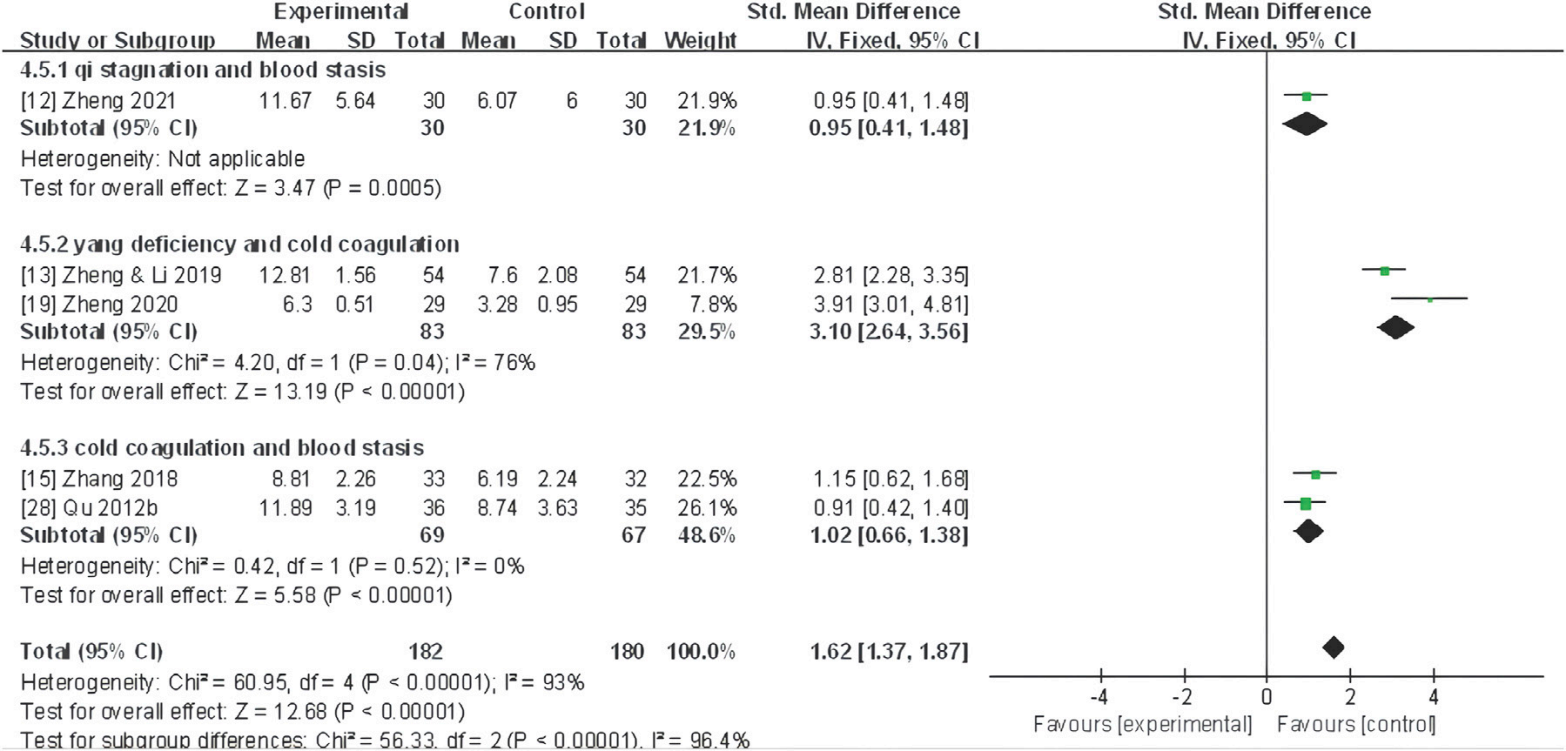
Forest plot for TCM syndrome scale of CHF subgroup analysis on different TCM patterns.

Further subgroup analyses based on different dysmenorrhea types and TCM patterns demonstrated that a significant decrease in the TCM syndrome score in four trials (Qu, 2012; Zhang, 2018; Zheng, 2020b; Zheng, 2021) of primary dysmenorrhea (MD 3.02, 95% CI: 2.66 to 3.38, *P*< 0.00001), one trial (Zheng and Li, 2019) of secondary dysmenorrhea (MD 5.21, 95% CI: 4.52 to 5.90, *P* < 0.00001) (Figure 9), as well as one trial (Zheng, 2021) of TCM pattern of qi stagnation and blood stasis in (SMD 0.95, 95% CI: 0.41 to 1.48, *P* < 0.001), two trials (Zheng and Li, 2019; Zheng, 2020a) of yang deficiency and cold coagulation (SMD 3.10, 95% CI: 2.64 to 3.56, *P* < 0.00001), and two trials (Qu, 2012; Zhang, 2018) of cold coagulation and blood stasis (SMD 1.02, 95% CI: 0.66 to 1.38, *P* < 0.00001) (Figure 10).

#### Adverse events

Four studies (Qu and Li, 2012; Lei and Liu, 2013; Ye and Xing, 2017a; Ye and Xing, 2017b) addressed the concern of adverse events, and two of which (Lei and Liu, 2013; Ye and Xing, 2017a) assessed the safety with blood, urine, and stool routine tests, as well as hepatic and renal function assessments. No adverse events were recorded in the CHF adjunctive treatment group.

#### Follow-up assessment

Five studies (Qu, 2012; Liu et al., 2018; Yang, 2018; Yuan et al., 2018; Zheng, 2020b) reported follow-up data over a 3-month span. Narratively, the CHF adjunctive treatment presented an optimal sustainable therapeutic benefit, as evident by the enhanced total effective rate (RR 1.34, 95% CI: 1.11 to 1.63, *P*< 0.01) in two trials (Yuan et al., 2018; Zheng, 2020a) and a diminished recurrence rate (RR 0.19, 95% CI: 0.09 to 0.39, *P*< 0.0001) in three trials (Qu, 2012; Liu et al., 2018; Yang, 2018) when compared with the control group (Supplementary Figures S3, S4).

### Publication bias assessment

Funnel plots were employed to evaluate the potential publication bias. The resultant plots for the total effective rate demonstrated an asymmetric distribution, suggesting a possibility of publication bias (Supplementary Figure S5). However, this potentiality was offset by the value of Egger’s test (*P* > 0.05), indicating the likelihood of publication bias was not evident.

### Sensitivity analysis

Sensitivity analyses were conducted for the total effective rate, VAS, and TCM syndrome scale. The results revealed that excluding any individual study from each outcome did not significantly alter the aggravated effect, indicating the stability and robustness of the pooled results.

## Discussion

Dysmenorrhea remains a predominant public health concern that impairs women’s quality of life, academic performance, and work productivity. Despite considerable research efforts, its complex pathomechanisms underlying are not yet fully deciphered. Beyond conventional pharmacological solutions, the medical community has gradually well-recognized the importance and promise of complementary and alternative interventions (Sosorburam et al., 2019; Su et al., 2021). Previous studies indicate the potential benefits of CHF in mitigating dysmenorrhea, yet comprehensive evidence remains limited. To the best of our knowledge, this is the first systematical review and meta-analysis to evaluate the effectiveness and safety of CHF as an adjunctive therapy for the management of dysmenorrhea.

The findings of the present meta-analysis suggested that CHF therapy could significantly enhance the total effective rate and reduce the VAS, CMSS, symptom score and TCM syndrome scale, consolidating its potential as an effective adjunctive intervention for patients suffered from dysmenorrhea. Furthermore, it displayed fewer adverse events and optimal sustainable long-term therapeutic benefits. The desirable clinical outcomes of CHF on dysmenorrhea are attributable to multi-factors. In the TCM paradigm, the feet are corresponded to different internal organs and regions of human body via specific channels and acupuncture points, and the absorption of Chinese herbs through skin and mucosa may act on these channels and acupuncture points, potentially alleviating visceral pain (Matos et al., 2021). Moreover, the thermal effect of footbaths may improve microcirculation and skin permeability, facilitating the assimilation of the active ingredients in the herbal concoctions (Zheng et al., 2019; Fu et al., 2020).

Moreover, the results of subgroup analysis based on different dysmenorrhea types and TCM patterns showed that CHF yielded a significant improvement in the total effective rate, symptom scores, and TCM syndrome scale for patients with either primary or secondary dysmenorrhea associated with qi stagnation and blood stasis, yang deficiency and cold coagulation, or cold coagulation and blood stasis. In the therapeutic framework of TCM, pattern differentiation serves as the foundation for the therapeutic interventions (Zheng, 2020b; Li X. et al., 2023). Dysmenorrhea is generally divided into two pathological categories, namely, Excesses of “pain due to obstruction” and Deficiency of “pain due to lack of nourishment.” The former is primarily arising from the blockage of qi and blood circulation due to internal and external pathogenic factors, such as cold, dampness, and heat, with cold-induced blockage being most notably prevalent. For relief, patients with “cold womb” are advised to expel cold and remove qi stagnation and blood stasis to relieve pain. While the latter is often caused by deficiency of Qi, blood, yin or yang, necessitating a focus on tonification and replenishment to address the deficiencies and nourish the “withered womb” (Sosorburam et al., 2019; Dong et al., 2022; Wu L.-J. et al., 2023). This meta-analysis incorporating 1,484 dysmenorrhea patients, either primary or secondary, identified cold coagulation, blood stasis, qi stagnation, and yang deficiency as prevalent etiopathogenesis. Accordingly, such well-recognized Chinese herbal prescriptions as *WenJing Tang* and *Shaofu Zhuyu Tang* are recommended for CHF to ensure the optimal clinical outcomes, as specified in Tables 2, 3. This also underscores the critical role of accurate pattern differentiation for CHF to achieve significant improvements in managing dysmenorrhea.

In addition, current insight into dysmenorrhea underscores its complex etiopathogenesis involving multiple factors, such as vasopressin, oxytocin, calcium, oxidative stress, inflammation, and nitric oxide, with prostaglandins (PGs), synthesized from arachidonic acid via the cyclooxygenase (COX) pathway, playing a pivotal role (Jabbour et al., 2006; Tu and Hellman, 2021; Snipe et al., 2024; Xiao et al., 2024). A further analysis demonstrates that 51 different Chinese herbs were employed in the 18 CHF prescriptions for dysmenorrhea in this meta-analysis, and 17 of which were identified as frequently used ingredients (frequency≥5 times), such as Chuanxiong Rhizoma (*Ligusticum chuanxiong Hort*) (n = 14), Angelicae Sinensis Radix [Dang Gui (Angelica sinensis (Oliv.) Diels) (n = 14), Euodiae Fructus [*Euodia rutaecarpa (Juss.) Benth*] (n = 13), Corydalis Rhizoma (*Corydalis yanhusuo W.T. Wang*) (n = 11), Paeoniae Radix Rubra (*Paeonia lactiflora Pall.*) (n = 8), Cinnamomi Ramulus [Cinnamomum cassia (L.) J. Presl] (n = 8), and Spatholobi Caulis (*Spatholobus suberectus Dunn*) (n = 8). These herbs are well-recognized for their pharmacologic effects of analgesia, spasmolysis, microcirculation, anti-inflammation, vasodilatation, and neuroprotection, as documented in Table 4. Experiments have also indicated the mechanism underlying their therapeutic effects on dysmenorrhea may attribute to modulate oestradiol, arginine vasopression, oxytocin and its receptor, PGE_2_ and PGF_2α_ expression; inhibit calcium channel, nuclear factor-κB(NF-κB), NF-κB/p38, mitogenactivated protein kinase, and COX-2; elevate nitric oxide and its synthetase; downregulate oxytocin, vasopressin, endothelin-1, malondialdehyde, superoxide, interleukin-6 (IL-6), IL-1β, monocyte chemotactic protein 1, inducible nitric oxide synthase, tumor necrosis factor-2α, whole blood viscosity, and plasma viscosity (Zhang et al., 2016; Sosorburam et al., 2019; Shao et al., 2020; Tan et al., 2020; Liu et al., 2021a; Dong et al., 2022; Mo et al., 2022; Wu T. et al., 2023; Cai and Feng, 2023; Li M. et al., 2024).

Although this meta-analysis assessed the effectiveness and safety of CHF as a supplementary treatment for dysmenorrhea, there are several limitations: 1) the small sample size of some studies might overrate the perceived effectiveness and undermine outcome reliability; 2) the inherent characteristics of CHF made blinding and allocation concealment unfeasible, potentially resulting in overestimated therapeutic benefits; 3) notable heterogeneity was presented in the aggravated results of symptom score and TCM syndrome scale, which might attribute to diverse efficacy criteria, differences in CHF formulation, and inconsistencies in treatment durations, temperatures, and immersion depths across studies. However, subgroup analysis was infeasible due to the limited number of studies, potentially compromising result accuracy and applicability; 4) the methodological quality of some studies was suboptimal and might cause an overestimated therapeutic effect; and 5) despite no language limitation for inclusion, all sourced publication were in Chinese, and the funnel plot implied the slight possibility of publication bias. Given these limitations, more well-designed, high-quality, large-sample sized RCTs are warranted to consolidate confidence in the therapeutic benefits of CHF for dysmenorrhea. Future research should also aim to evaluate the holistic impact of CHF on dysmenorrhea patients in such variables as over-all quality of life and sleep quantity and quality.

## Conclusion

In conclusion, this study suggests that Chinese herbal footbaths may serve as a promising and safe adjuvant therapy for dysmenorrhea management. However, the limited data and variable methodological quality of the included studies necessitate a cautious interpretation of these findings. Further verification with more well-designed high-quality multicenter RCTs of large sample size are warranted.

## Data Availability

The original contributions presented in the study are included in the article/Supplementary Material, further inquiries can be directed to the corresponding authors.

## Supplementary material

Supplementary Figure S1 Forest plot for VAS score of adjunctive CHF therapy versus control group. CHF, Chinese herbal footbaths; VAS, visual analogue scale.

Supplementary Figure S2 Forest plot for CMSS score of adjunctive CHF therapy versus control group. CHF, Chinese herbal footbaths; CMSS, the Cox Menstrual Symptom Scale.

Supplementary Figure S3 Forest plot for the total effective rate of follow-up.

Supplementary Figure S4 Forest plot for the recurrence rate of follow-up.

Supplementary Figure S5 Funnel plots assessing publication bias for the total effective rate.
