# Supplementary figures and images for: Effectiveness and safety of Chinese herbal footbaths as an adjuvant therapy for dysmenorrhea: a systematic review and meta-analysis
Source: Front Pharmacol. 2024 Aug 5;15:1397359. doi: 10.3389/fphar.2024.1397359 (PMC11331266; doi:10.3389/fphar.2024.1397359)

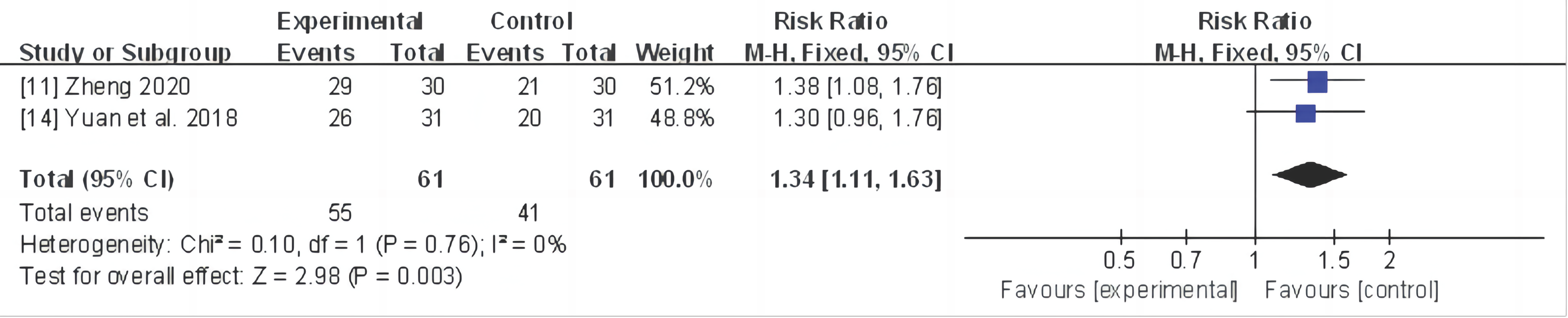

Supplement: Supplementary file 1 [file Image3.TIFF]

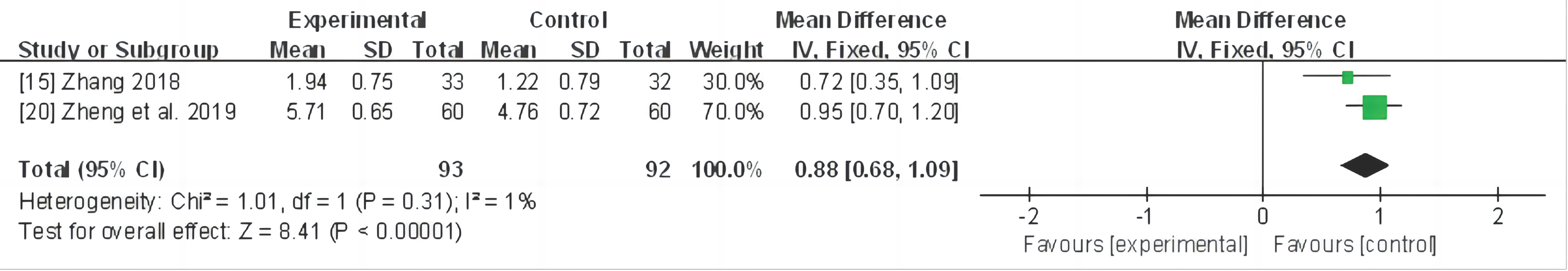

Supplement: Supplementary file 2 [file Image1.TIFF]

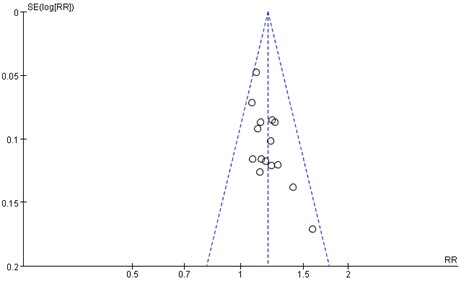

Supplement: Supplementary file 3 [file Image5.TIFF]

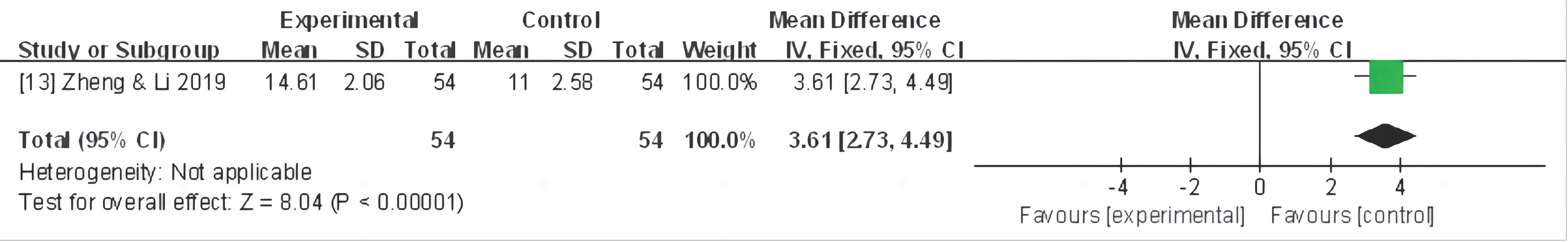

Supplement: Supplementary file 4 [file Image2.TIFF]

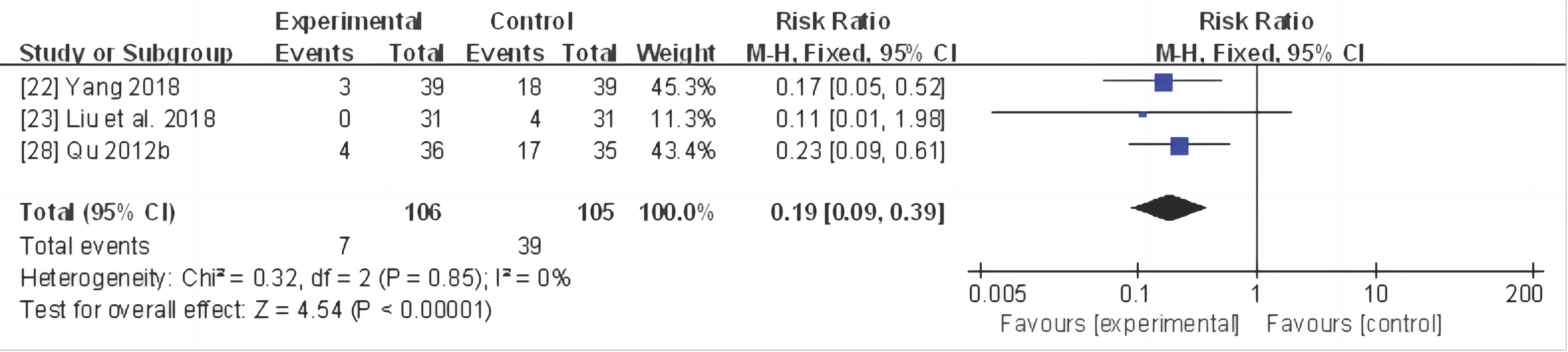

Supplement: Supplementary file 5 [file Image4.TIFF]
